# Personal

**Published:** 1899-07

**Authors:** 


### PERSONAL.

DR. HORACE M. PAINE, of Albany, one of the most prominent homeopathic physicians in the country, was recently tendered a complimentary dinner at the Fort Schuyler Club, Utica, N.Y., by a number of his professional confréres, the occasion being the fiftieth anniversary of his graduation in medicine.

---

DR. NELSON W. WILSON has resigned from the medical department of the United States Army after a period of nine months' service as assistant surgeon at Fort Porter, and has resumed private practice. He is associated with Dr. W. H. Heath at the latter's office, No. 131 Franklin street.

---

DR. MAX C. BREUER, of Buffalo, sailed for Europe on June 1, 1899, to spend three or four months in the hospitals of Breslau University. He was accompanied by Mrs. Breuer. Dr. Breuer will return and take up his professional work in this city early in the autumn.

---

DR. M. ALLEN STARR, of New York, received the degree of LL. D. from Princeton University at its last commencement.

---

DR. NELSON G. RICHMOND, of Fredonia, recently took passage for Europe by the Hamburg-American line, where he will spend the summer in study and recreation.

Dr. and Mrs. Frank W. Abbott and Frank W. Abbott, Jr., have arrived in England. They sailed from Boston on the New England, May 31, 1899.

---

Dr. F. Park Lewis sailed on June 21, 1899, on the Fuerst Bismarck, with Dr. Knapp, of New York, for Hamburg. They will be absent until September.

---

Dr. Alvin A. Hubbell will sail for Europe July 19th, and during his sojourn there will attend the International Otological Congress, at London, August 8–11th, and the International Ophthalmological Congress, at Utrecht, Holland, August 14-18th. He will return early in September.

